# *catena*-Poly[[aqua­dioxidouranium(VI)]-μ_3_-4,4′-oxydibenzoato]

**DOI:** 10.1107/S1600536810010639

**Published:** 2010-03-27

**Authors:** Wei Wang, Dao-Jun Zhang, Yong Fan, Tian-You Song, Ping Zhang

**Affiliations:** a State Key Laboratory of Inorganic Synthesis and Preparative Chemistry, College of Chemistry, Jilin University, Changchun 130012, People’s Republic of China

## Abstract

The title compound, [UO_2_(C_14_H_8_O_5_)(H_2_O)]_*n*_, is a polymeric UO_2_ complex bridged by 4,4′-oxydibenzoate ligands. One carboxyl­ate group of the bridging ligand chelates a uranyl cation while the other carboxyl­ate group of the ligand bridges two other two uranyl cations, forming a double-chain polymeric structure. The central U^VI^ atom is seven-coordin­ated in a distorted UO_7_ penta­gonal-bipyramidal geometry. In the crystal structure, O—H⋯O hydrogen bonding links the polymeric chains into a three-dimensional supra­molecular framework. Within the bridging ligand, the two benzene rings are oriented at a dihedral angle of 59.0 (2)°.

## Related literature

For the potential applications of porous uranyl-organic frameworks, see: Thuéry & Masci (2008 ▶); Cahill *et al.* (2007 ▶); Masci & Thuéry (2008 ▶). For a related structure, see: Yu *et al.* (2004 ▶).
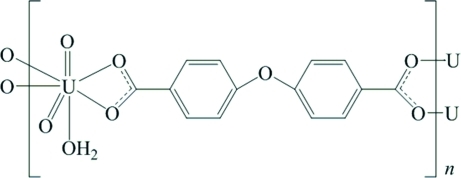


## Experimental

### 

#### Crystal data

[U(C_14_H_8_O_5_)O_2_(H_2_O)]
*M* *_r_* = 544.25
Monoclinic, 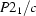

*a* = 16.0160 (6) Å
*b* = 8.8832 (3) Å
*c* = 10.1952 (4) Å
β = 95.736 (1)°
*V* = 1443.24 (9) Å^3^
*Z* = 4
Mo *K*α radiation
μ = 11.29 mm^−1^
*T* = 296 K
0.19 × 0.18 × 0.17 mm

#### Data collection

Rigaku R-AXIS RAPID diffractometer
Absorption correction: multi-scan (*ABSCOR*; Higashi, 1995 ▶) *T*_min_ = 0.135, *T*_max_ = 0.147
10376 measured reflections
3606 independent reflections
3188 reflections with *I* > 2σ(*I*)
*R*_int_ = 0.020

#### Refinement

*R*[*F* ^2^ > 2σ(*F* ^2^)] = 0.019
*wR*(*F* ^2^) = 0.064
*S* = 1.17
3606 reflections
208 parameters
H-atom parameters constrained
Δρ_max_ = 0.74 e Å^−3^
Δρ_min_ = −0.88 e Å^−3^

### 

Data collection: *RAPID-AUTO* (Rigaku, 1998 ▶); cell refinement: *RAPID-AUTO*; data reduction: *CrystalClear* (Rigaku/MSC, 2002 ▶); program(s) used to solve structure: *SHELXS97* (Sheldrick, 2008 ▶); program(s) used to refine structure: *SHELXL97* (Sheldrick, 2008 ▶); molecular graphics: *ORTEP-3 for Windows* (Farrugia, 1997 ▶); software used to prepare material for publication: *WinGX* (Farrugia, 1999 ▶).

## Supplementary Material

Crystal structure: contains datablocks I, global. DOI: 10.1107/S1600536810010639/xu2712sup1.cif

Structure factors: contains datablocks I. DOI: 10.1107/S1600536810010639/xu2712Isup2.hkl

Additional supplementary materials:  crystallographic information; 3D view; checkCIF report

## Figures and Tables

**Table 1:** Selected bond lengths (Å)

U1—O1^i^	2.337 (3)
U1—O2	2.315 (3)
U1—O4^ii^	2.407 (3)
U1—O5^ii^	2.437 (3)
U1—O6	1.759 (3)
U1—O7	1.765 (3)
U1—O8	2.461 (3)

Symmetry codes: (i) 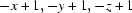; (ii) 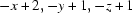.

**Table 2:** Hydrogen-bond geometry (Å, °)

*D*—H⋯*A*	*D*—H	H⋯*A*	*D*⋯*A*	*D*—H⋯*A*
O8—H8⋯O7^iii^	0.85	2.05	2.812 (4)	150
O8—H11⋯O2^iv^	0.85	2.37	3.083 (4)	142

Symmetry codes: (iii) 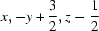; (iv) 
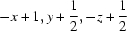
.

**Fig. 1.**
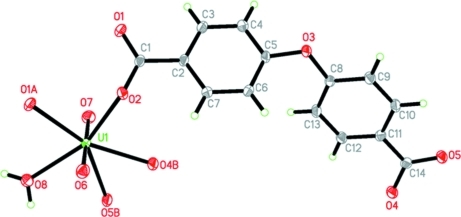
Thermal ellipsoid plot of a portion of the title compound, showing the atom-labelling scheme. Displacement ellipsoids are drawn at the 30% probability level [symmetry codes (A): -x + 1, -y + 1, -z + 1; (B): -x + 2, -y + 1, -z + 1].

**Fig. 2.**
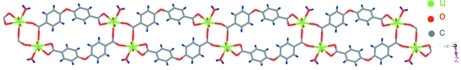
View of one-dimensional double chain structure of the title compound.

**Fig. 3.**
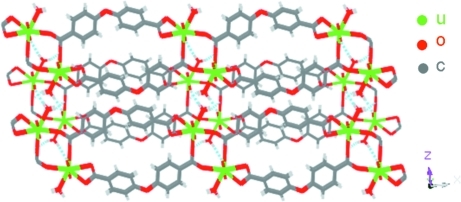
View of three-dimensional supramolecular structure with hydrogen bond interactions shown as dashed lines.

## supplementary crystallographic information

### Comment

Recently the design and synthesis of porous uranyl-organic frameworks (UOFs) have received rather considerable attention due to their potential applications (Thuéry & Masci, 2008; Cahill *et al.*, 2007; Masci & Thuéry, 2008; Yu *et al.*, 2004; Chen *et al.*, 2003). However the coordination polymers of uranyl with 4,4'-oxydibenzoate ligand (oba) have not been reported to our knowledge. We are currently interested in using oba as a bridging ligand to build novel UOFs. Herein we report a novel UOF of [UO_2_(oba)(H_2_O)]_n_.

The compound possesses a 1-D double chain architecture, in which the asymmetric unit consists of one UO_2_^2+^ cation, one oxy-bis(benzoate) anion and one coordinated water molecule. The central U atom is seven-coordinated in a UO_7_ pentagonal-bipyramidal geometry by four O atoms from three different oba^2-^ ligands and one water O atom, and other two O atoms are oxo groups in axial positions (Fig. 1). The coordinate bond distances are listed in Table 1, which are comparable to the related uranyl carboxylate compound (Yu *et al.*, 2004). The terminal groups of the oba ligand coordinate to three U centers to form the 1D double chain framework (Fig. 2). The O—H···O hydrogen bonding links the polymeric chains into 3D supramolecular structure (Table 2 and Fig. 3).

### Experimental

For the preparation of the title compound, a mixture of uranium oxynitrate hexahydrate (0.1025 g, 0.21 mmol), 4,4'-oxybis(benzoic acid) (0.1092 g, 0.42 mmol), potassium hydroxide (0.0470 g, 0.84 mmol) and 6 mL water was sealed in a 23 mLstainless steel reactor with a Teflon liner. The reaction system was heated at 423 K for 48 h, and then cooled slowly to room temperature. A large number of pale-yellow crystals of the title compound were obtained and collected by filtration, washed with water and dried in air.

### Refinement

H atoms bound to C atoms were placed in calculated positions and treated as riding on their parent atoms, with C—H = 0.93 Å (aromatic) and with U_iso_(H) = 1.2U_eq_(C). Water H atoms were initially located in a difference Fourier map, but they were treated as riding on their parent atoms with O—H = 0.85 Å and with U_iso_(H) = 1.5U_eq_(O).

### Crystal data

**Table:**

[U(C_14_H_8_O_5_)O_2_(H_2_O)]	*F*(000) = 1000
*M**_r_* = 544.25	*D*_x_ = 2.505 Mg m^−^^3^
Monoclinic, *P*2_1_/*c*	Mo *K*α radiation, λ = 0.71073 Å
Hall symbol: -P 2ybc	Cell parameters from 4425 reflections
*a* = 16.0160 (6) Å	θ = 2.5–25.0°
*b* = 8.8832 (3) Å	µ = 11.29 mm^−^^1^
*c* = 10.1952 (4) Å	*T* = 296 K
β = 95.736 (1)°	Block, yellow
*V* = 1443.24 (9) Å^3^	0.19 × 0.18 × 0.17 mm
*Z* = 4	

### Data collection

**Table:**

Rigaku R-AXIS RAPID diffractometer	3606 independent reflections
Radiation source: fine-focus sealed tube	3188 reflections with *I* > 2σ(*I*)
graphite	*R*_int_ = 0.020
ω scan	θ_max_ = 28.4°, θ_min_ = 1.3°
Absorption correction: multi-scan (*ABSCOR*; Higashi, 1995)	*h* = −21→20
*T*_min_ = 0.135, *T*_max_ = 0.147	*k* = −11→11
10376 measured reflections	*l* = −13→11

### Refinement

**Table:**

Refinement on *F*^2^	Primary atom site location: structure-invariant direct methods
Least-squares matrix: full	Secondary atom site location: difference Fourier map
*R*[*F*^2^ > 2σ(*F*^2^)] = 0.019	Hydrogen site location: inferred from neighbouring sites
*wR*(*F*^2^) = 0.064	H-atom parameters constrained
*S* = 1.17	*w* = 1/[σ^2^(*F*_o_^2^) + (0.0365*P*)^2^] where *P* = (*F*_o_^2^ + 2*F*_c_^2^)/3
3606 reflections	(Δ/σ)_max_ = 0.002
208 parameters	Δρ_max_ = 0.74 e Å^−^^3^
0 restraints	Δρ_min_ = −0.88 e Å^−^^3^

### Special details

**Table:**

Geometry. All e.s.d.'s (except the e.s.d. in the dihedral angle between two l.s. planes) are estimated using the full covariance matrix. The cell e.s.d.'s are taken into account individually in the estimation of e.s.d.'s in distances, angles and torsion angles; correlations between e.s.d.'s in cell parameters are only used when they are defined by crystal symmetry. An approximate (isotropic) treatment of cell e.s.d.'s is used for estimating e.s.d.'s involving l.s. planes.
Refinement. Refinement of *F*^2^ against ALL reflections. The weighted *R*-factor *wR* and goodness of fit *S* are based on *F*^2^, conventional *R*-factors *R* are based on *F*, with *F* set to zero for negative *F*^2^. The threshold expression of *F*^2^ > σ(*F*^2^) is used only for calculating *R*-factors(gt) *etc*. and is not relevant to the choice of reflections for refinement. *R*-factors based on *F*^2^ are statistically about twice as large as those based on *F*, and *R*- factors based on ALL data will be even larger.

### Fractional atomic coordinates and isotropic or equivalent isotropic displacement parameters (Å^2^)

**Table:**

	*x*	*y*	*z*	*U*_iso_*/*U*_eq_	
U1	0.586399 (8)	0.547362 (15)	0.296990 (13)	0.02255 (6)	
O1	0.55538 (19)	0.4926 (4)	0.6780 (3)	0.0334 (6)	
O4	1.2633 (2)	0.4586 (4)	0.6570 (3)	0.0393 (8)	
O5	1.31269 (18)	0.3423 (4)	0.8388 (3)	0.0366 (7)	
C14	1.2507 (3)	0.3891 (5)	0.7634 (4)	0.0287 (8)	
C11	1.1633 (2)	0.3682 (4)	0.7976 (4)	0.0259 (8)	
C12	1.0998 (3)	0.4558 (4)	0.7347 (4)	0.0293 (9)	
H12	1.1117	0.5218	0.6683	0.035*	
C10	1.1455 (3)	0.2688 (5)	0.8968 (4)	0.0303 (8)	
H10	1.1882	0.2115	0.9408	0.036*	
C13	1.0187 (3)	0.4458 (4)	0.7699 (4)	0.0316 (9)	
H13	0.9765	0.5067	0.7294	0.038*	
C9	1.0644 (2)	0.2558 (5)	0.9295 (4)	0.0308 (8)	
H9	1.0521	0.1877	0.9941	0.037*	
C8	1.0012 (2)	0.3435 (5)	0.8665 (4)	0.0269 (8)	
C1	0.6145 (3)	0.4466 (4)	0.6168 (4)	0.0251 (8)	
C2	0.6980 (2)	0.4239 (4)	0.6891 (4)	0.0243 (8)	
C7	0.7623 (2)	0.3572 (5)	0.6279 (4)	0.0279 (8)	
H7	0.7535	0.3328	0.5389	0.033*	
C6	0.8389 (3)	0.3266 (5)	0.6969 (4)	0.0304 (9)	
H6	0.8814	0.2807	0.6555	0.037*	
C5	0.8514 (2)	0.3659 (5)	0.8298 (4)	0.0273 (8)	
C4	0.7882 (3)	0.4337 (5)	0.8920 (4)	0.0321 (9)	
H4	0.7974	0.4596	0.9806	0.038*	
C3	0.7115 (3)	0.4629 (4)	0.8222 (4)	0.0307 (9)	
H3	0.6690	0.5084	0.8638	0.037*	
O3	0.92367 (17)	0.3279 (4)	0.9105 (3)	0.0370 (7)	
O2	0.60500 (19)	0.4162 (4)	0.4939 (3)	0.0345 (7)	
O7	0.5825 (2)	0.7188 (3)	0.3837 (3)	0.0359 (7)	
O6	0.58900 (19)	0.3749 (3)	0.2127 (3)	0.0350 (7)	
O8	0.50947 (19)	0.6486 (3)	0.0952 (3)	0.0369 (7)	
H11	0.4613	0.6883	0.0787	0.055*	
H8	0.5375	0.6547	0.0288	0.055*	

### Atomic displacement parameters (Å^2^)

**Table:**

	*U*^11^	*U*^22^	*U*^33^	*U*^12^	*U*^13^	*U*^23^
U1	0.01670 (9)	0.02981 (9)	0.02128 (9)	0.00041 (5)	0.00266 (6)	−0.00035 (5)
O1	0.0196 (15)	0.0439 (16)	0.0374 (17)	0.0045 (13)	0.0064 (12)	0.0048 (14)
O4	0.0205 (16)	0.066 (2)	0.0309 (16)	−0.0022 (14)	0.0011 (12)	0.0114 (14)
O5	0.0197 (14)	0.0566 (19)	0.0330 (15)	−0.0005 (14)	0.0010 (12)	0.0132 (14)
C14	0.022 (2)	0.037 (2)	0.0282 (19)	−0.0036 (17)	0.0037 (15)	−0.0004 (17)
C11	0.0195 (18)	0.032 (2)	0.0260 (18)	−0.0040 (16)	0.0004 (14)	−0.0040 (16)
C12	0.023 (2)	0.036 (2)	0.029 (2)	−0.0037 (16)	0.0002 (16)	0.0052 (16)
C10	0.023 (2)	0.035 (2)	0.031 (2)	0.0035 (17)	−0.0028 (15)	0.0052 (17)
C13	0.022 (2)	0.038 (2)	0.035 (2)	0.0007 (16)	−0.0016 (17)	0.0073 (17)
C9	0.022 (2)	0.038 (2)	0.032 (2)	−0.0036 (17)	0.0006 (15)	0.0086 (17)
C8	0.0161 (18)	0.040 (2)	0.0235 (18)	−0.0034 (16)	−0.0017 (14)	−0.0002 (16)
C1	0.0184 (19)	0.0268 (19)	0.030 (2)	−0.0022 (14)	0.0024 (15)	0.0066 (15)
C2	0.0156 (18)	0.0284 (19)	0.029 (2)	−0.0013 (14)	0.0012 (15)	0.0050 (15)
C7	0.024 (2)	0.036 (2)	0.0233 (18)	0.0026 (17)	0.0018 (15)	−0.0009 (16)
C6	0.0204 (19)	0.044 (2)	0.027 (2)	0.0069 (17)	0.0026 (15)	−0.0029 (17)
C5	0.0165 (18)	0.038 (2)	0.0270 (18)	−0.0005 (16)	−0.0020 (14)	0.0056 (16)
C4	0.025 (2)	0.044 (2)	0.026 (2)	−0.0015 (18)	−0.0005 (16)	−0.0021 (17)
C3	0.023 (2)	0.040 (2)	0.030 (2)	0.0051 (16)	0.0025 (17)	−0.0051 (16)
O3	0.0169 (14)	0.066 (2)	0.0278 (14)	0.0009 (14)	−0.0014 (11)	0.0103 (14)
O2	0.0238 (16)	0.0517 (17)	0.0270 (15)	0.0005 (13)	−0.0015 (12)	0.0113 (13)
O7	0.0363 (17)	0.0354 (16)	0.0362 (16)	0.0002 (13)	0.0045 (13)	−0.0099 (13)
O6	0.0298 (16)	0.0351 (16)	0.0399 (17)	0.0026 (13)	0.0024 (12)	−0.0072 (13)
O8	0.0334 (17)	0.0473 (17)	0.0298 (15)	0.0056 (14)	0.0027 (12)	0.0101 (13)

### Geometric parameters (Å, °)

**Table:**

U1—O1^i^	2.337 (3)	C10—H10	0.9300
U1—O2	2.315 (3)	C13—C8	1.389 (6)
U1—O4^ii^	2.407 (3)	C13—H13	0.9300
U1—O5^ii^	2.437 (3)	C9—C8	1.384 (5)
U1—O6	1.759 (3)	C9—H9	0.9300
U1—O7	1.765 (3)	C8—O3	1.369 (5)
U1—O8	2.461 (3)	C1—O2	1.275 (5)
U1—C14^ii^	2.798 (4)	C1—C2	1.475 (5)
O1—C1	1.254 (5)	C2—C7	1.389 (5)
O1—U1^i^	2.337 (3)	C2—C3	1.396 (6)
O4—C14	1.282 (5)	C7—C6	1.379 (5)
O4—U1^ii^	2.407 (3)	C7—H7	0.9300
O5—C14	1.263 (5)	C6—C5	1.395 (5)
O5—U1^ii^	2.437 (3)	C6—H6	0.9300
C14—C11	1.488 (5)	C5—C4	1.385 (6)
C14—U1^ii^	2.798 (4)	C5—O3	1.393 (4)
C11—C12	1.386 (6)	C4—C3	1.381 (6)
C11—C10	1.393 (5)	C4—H4	0.9300
C12—C13	1.384 (6)	C3—H3	0.9300
C12—H12	0.9300	O8—H11	0.8499
C10—C9	1.377 (6)	O8—H8	0.8500
			
O6—U1—O7	178.90 (14)	C13—C12—H12	119.7
O6—U1—O2	88.75 (12)	C11—C12—H12	119.7
O7—U1—O2	90.45 (12)	C9—C10—C11	119.6 (4)
O6—U1—O1^i^	89.53 (13)	C9—C10—H10	120.2
O7—U1—O1^i^	89.62 (13)	C11—C10—H10	120.2
O2—U1—O1^i^	82.59 (10)	C12—C13—C8	119.2 (4)
O6—U1—O4^ii^	90.22 (13)	C12—C13—H13	120.4
O7—U1—O4^ii^	90.34 (13)	C8—C13—H13	120.4
O2—U1—O4^ii^	77.30 (10)	C10—C9—C8	120.3 (4)
O1^i^—U1—O4^ii^	159.90 (11)	C10—C9—H9	119.8
O6—U1—O5^ii^	91.33 (12)	C8—C9—H9	119.8
O7—U1—O5^ii^	89.77 (13)	O3—C8—C9	115.9 (3)
O2—U1—O5^ii^	131.32 (10)	O3—C8—C13	123.5 (4)
O1^i^—U1—O5^ii^	146.09 (10)	C9—C8—C13	120.5 (4)
O4^ii^—U1—O5^ii^	54.01 (10)	O1—C1—O2	122.4 (4)
O6—U1—O8	86.79 (12)	O1—C1—C2	119.2 (4)
O7—U1—O8	93.66 (12)	O2—C1—C2	118.4 (4)
O2—U1—O8	156.87 (10)	C7—C2—C3	119.5 (4)
O1^i^—U1—O8	74.70 (10)	C7—C2—C1	120.6 (4)
O4^ii^—U1—O8	125.36 (10)	C3—C2—C1	119.8 (4)
O5^ii^—U1—O8	71.51 (10)	C6—C7—C2	121.1 (4)
O6—U1—C14^ii^	90.00 (13)	C6—C7—H7	119.4
O7—U1—C14^ii^	90.93 (13)	C2—C7—H7	119.4
O2—U1—C14^ii^	104.52 (11)	C7—C6—C5	118.7 (4)
O1^i^—U1—C14^ii^	172.86 (11)	C7—C6—H6	120.6
O4^ii^—U1—C14^ii^	27.22 (11)	C5—C6—H6	120.6
O5^ii^—U1—C14^ii^	26.82 (10)	C4—C5—O3	116.0 (3)
O8—U1—C14^ii^	98.17 (11)	C4—C5—C6	120.9 (4)
C1—O1—U1^i^	142.7 (3)	O3—C5—C6	122.9 (4)
C14—O4—U1^ii^	93.6 (2)	C3—C4—C5	119.9 (4)
C14—O5—U1^ii^	92.7 (2)	C3—C4—H4	120.0
O5—C14—O4	119.6 (4)	C5—C4—H4	120.0
O5—C14—C11	121.1 (3)	C4—C3—C2	119.9 (4)
O4—C14—C11	119.3 (4)	C4—C3—H3	120.1
O5—C14—U1^ii^	60.5 (2)	C2—C3—H3	120.1
O4—C14—U1^ii^	59.2 (2)	C8—O3—C5	120.7 (3)
C11—C14—U1^ii^	175.5 (3)	C1—O2—U1	137.5 (3)
C12—C11—C10	119.9 (4)	U1—O8—H11	133.3
C12—C11—C14	119.1 (4)	U1—O8—H8	115.4
C10—C11—C14	121.0 (4)	H11—O8—H8	110.9
C13—C12—C11	120.5 (4)		

Symmetry codes: (i) −*x*+1, −*y*+1, −*z*+1; (ii) −*x*+2, −*y*+1, −*z*+1.

### Hydrogen-bond geometry (Å, °)

**Table:**

*D*—H···*A*	*D*—H	H···*A*	*D*···*A*	*D*—H···*A*
O8—H8···O7^iii^	0.85	2.05	2.812 (4)	150
O8—H11···O2^iv^	0.85	2.37	3.083 (4)	142

Symmetry codes: (iii) *x*, −*y*+3/2, *z*−1/2; (iv) −*x*+1, *y*+1/2, −*z*+1/2.
